# Recent Advances in Engineering the Stem Cell Microniche in 3D

**DOI:** 10.1002/advs.201800448

**Published:** 2018-06-13

**Authors:** Min Bao, Jing Xie, Wilhelm T. S. Huck

**Affiliations:** ^1^ Institute for Molecules and Materials Radboud University Heyendaalseweg 135 6525 AJ Nijmegen The Netherlands

**Keywords:** 3D cell cultures, cell geometries, dimensionality, mechanotransduction, microenvironments

## Abstract

Conventional 2D cell culture techniques have provided fundamental insights into key biochemical and biophysical mechanisms responsible for various cellular behaviors, such as cell adhesion, spreading, division, proliferation, and differentiation. However, 2D culture in vitro does not fully capture the physical and chemical properties of the native microenvironment. There is a growing body of research that suggests that cells cultured on 2D substrates differ greatly from those grown in vivo. This article focuses on recent progress in using bioinspired 3D matrices that recapitulate as many aspects of the natural extracellular matrix as possible. A range of techniques for the engineering of 3D microenvironment with precisely controlled biophysical and chemical properties, and the impact of these environments on cellular behavior, is reviewed. Finally, an outlook on future challenges for engineering the 3D microenvironment and how such approaches would further our understanding of the influence of the microenvironment on cell function is provided.

## 1 Introduction

In vivo, stem cells reside in a complex, specialized, and dynamic microenvironment, or “microniche.”^1^ Although these microenvironments are extremely diverse, they share a number of characteristic features of function and composition.^2^ The microenvironment serves as a structural support for cells, but also offers various biochemical (e.g., cell–cell contact, cell adhesion sites, and insoluble factors) and biophysical (e.g., topography, porosity, and rigidity) cues that together regulate cell behavior, including cell spreading, migration, differentiation, and self‐renewal.

**Figure 1:**
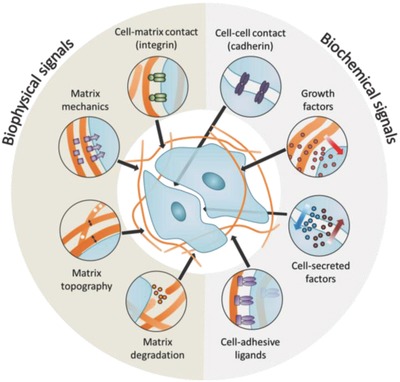
Niche interactions known to modulate stem cell phenotype. The biochemical composition, mechanical properties, and microstructure of the ECM are all known to modulate stem cell behavior, with optimal properties dependent on both the stem cell type of interest and the desired phenotypic output.

The extracellular matrix (ECM), a key constitutive part of the microniche, plays an essential role in regulating cell behavior,^3^ and supports cell or organ development, function, and repair. The physical properties of the ECM (topography, porosity, rigidity) all impact on biological functions that are related to cell spreading, division, migration, or tissue polarity. In addition, the ECM provides biochemical signaling cues that regulate cell phenotype (**Figure** **1**).

Stem cells, including pluripotent stem cells, embryonic stem cells (ESCs), mesenchymal stem cells (MSCs), hematopoietic stem cells, and neural stem cells, have been widely used for investigating fundamental interactions between cells and the ECM, and have potential applications in translational regenerative medicine or stem cell therapy. Thus, controlling stem cell fate (the ability to maintain the stemness, or to differentiate into different cell types) through engineered microniches is becoming particularly important in cell biology and tissue engineering field. Recently, numerous studies have shown that engineered microniches that mimic different aspects of the native stem cell niche can promote maintenance of stem cell quiescence (which is necessary for long‐term culture of stem cells to generate disease models),^4^ facilitate stem cell expansion (which is needed for stem cell delivery and stem cell therapy),^5^ and regulate stem cell differentiation (which can be used for tissue engineered constructs).^6^

In this review, we will discuss the role of the microniche in controlling cell function, with a specific emphasis on the importance on the role of the ECM. We will start with a short overview on different properties of the ECM that regulate cell fate, and then examine the differences between 2D and 3D cell culture. We will also provide an overview of the techniques used for investigating the interactions between ECM and stem cells in 3D, and discuss current advances toward designing 3D engineered niches.

## 2 The Stem Cell Microniche

The stem cell niche consists of a myriad of interacting components (Figure 1), which may include the ECM, other cells, growth factors, and heterologous cell types (e.g., endothelial cells). These components provide biophysical and biochemical inputs that regulate cell behavior such as adhesion, spreading, migration, division, self‐renewal, quiescence, and differentiation. This section reviews recent progress in studying the effect of different ECM properties on regulating cell fate determination and engineering approaches to control the stem cell microenvironment.

### 2.1 Extracellular Matrix Mechanics

The native ECM is a network of fibrillar proteins and polysaccharides that anchors cells within their specific microenvironment. Cells are mechanically coupled to the ECM through transmembrane proteins known as integrins.^7^ These integrins bind specific cell‐adhesive ligands presented by ECM proteins, connecting the ECM to the intracellular actin cytoskeleton. During cell spreading and growth, the ECM can be mechanically deformed and remodeled by cells,^8^ the mechanical properties of the ECM alter the ability of cells to generate tension, modulating cell spreading, nuclear shape, and intercellular signaling pathways. Different types of mechanics can influence cell behavior in different ways, including bulk stiffness, local stiffness, strain‐stiffening, and stress‐relaxation.

#### 2.1.1 Bulk Stiffness

**Figure 2:**
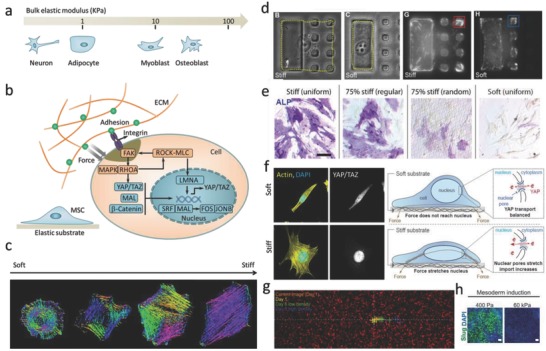
Bulk stiffness regulates stem cell fate. a) The differentiation of MSCs toward particular lineages is regulated by substrates with stiffness that is similar to native tissues. b) Mechanotransduction pathways inside cells regulate cell fates. c) Actin cytoskeleton organization depends on substrate stiffness. The different colors indicate different orientations of actin filaments. Reproduced with permission.^9^ Copyright 2015, Nature Publishing Group. d) A micropatterned platform that limits cells to a stiff region stimulates durotaxis. Reproduced with permission.^10^ Copyright 2014, National Academy of Sciences (United States). e) Spatially patterned matrix elasticity directs stem cell differentiation. Reproduced with permission.^11^ Copyright 2016, National Academy of Sciences (United States). f) Stiffness triggers nuclear YAP localization by regulating transport across nuclear pores. Reproduced with permission.^12^ Copyright 2017, Cell Press. g) Stiffness gradient affects cell migration, cells can migrate from soft to stiff. Reproduced with permission.^13^ Copyright 2017, National Academy of Sciences (United States). h) Stiffness determines embryonic stem cell differentiation. Reproduced with permission.^14^ Copyright 2016, Cell Press.

Substrate stiffness, typically characterized by the elastic or Young's modulus, has emerged as one of the most important mechanical features in controlling cell fate. This means that cells can sense the resistance of the substrate (typically a hydrogel) toward deformation. Modifications to the bulk stiffness of ECM‐coated hydrogels give rise to a range of responses in stem cells. On 2D substrates, mesenchymal stem cells typically show differentiation toward osteoblasts on stiff substrates while lineage selection on soft substrates favors adipocytes[[qv: 6a]] (**Figure** **2**a).

During mechanotransduction, mechanical stimuli, such as stretching, shear stress, or substrate rigidity, are converted into chemical signals that control cell fate.^15^ Key in this process are focal adhesions (FAs)^16^ and cell–cell interactions (involving, among others, β1‐integrin^7^ and E‐cadherin^17^), mechanosensors (such as talin^15^) and nuclear signaling elements (for example, yes‐associated protein (YAP)/transcriptional coactivator (TAZ)^18^ and lamin A/C^19^), which together act to modify protein and gene expression profiles (Figure 2b). Until now, substrates with stiffnesses ranging from a few hundred Pa to MPa have been prepared in a range of model substrates, including natural material such as chitosan, hyaluronic acid, gelatin, alginate, and agarose, or synthetic hydrogels such polyethylene glycol (PEG), poly(vinyl alcohol) (PVA), or polyacrylamide. Cells cultured on these hydrogels are responsive to the degree of stiffness by altering their adhesion, spreading, morphology, and migration characteristics. For instance, fibroblasts or endothelial cells cultured on a relatively stiff substrate (>2–3 kPa) display significant spreading and generate greater actin stress fibers compared with those on a relatively soft substrate (<2–3 kPa).^20^ The orientations of actin filaments strongly depend on substrate stiffness, with stiffer substrates can leading to more aligned actin filaments (Figure 2c).^9^ Cell spreading is also affected by stiffness, and by preparing a rigid domain of one large adhesive island, adjacent to a soft area of small adhesive islands grafted in an otherwise nonadhesive soft hydrogel, researchers have shown that cells spread and probe substrate stiffness by using filopodia extensions (Figure 2d).^10^ Matrix stiffness often shows local heterogeneities at different length scales within the natural niche.^11^, ^21^ Yang et al. fabricated a hydrogel with regions of spatially varied and distinct mechanics, and they found that hMSCs cultured on hydrogels with higher concentrations of stiff regions showed more spread, elongated cell morphologies, higher nuclear YAP localization, and higher osteoblast differentiation, indicating that local variations in the underlying substrate mechanical properties might regulate cell adhesion, spreading, and nuclear transcription effectors (Figure 2e).^11^ The effect of stiffness of cell function can often be related to the activity of certain nuclear transcription factors (YAP/TAZ),^18^ and it was shown recently that stiffer substrates give rise to nuclear flattening, thereby stretching nuclear pores, and reducing their mechanical resistance to molecular transport, and finally increasing YAP nuclear import and localization (Figure 2f).^12^ In addition, cell migration is also affected by stiffness.^13^, ^22^ When subjected to a stiffness gradient, cells display directed migration toward stiffer regions. The anisotropic mechanical properties lead to directional epithelial growth and trigger cells to migrate at the direction where the stiffness is larger, a behavior termed durotaxis, which is considered to contribute to the repair of tissue (Figure 2g).[[qv: 22a]] It has been shown that matrix stiffness also guides the spreading and differentiation of ESCs (Figure 2h),^14^ where softer substrates enhance mesoderm differentiation of human ESCs.

However, much of our knowledge about stiffness‐induced stem cell differentiation on 2D cell cultures cannot be directly translated to a 3D environment. For example, it was recently reported that hMSCs, encapsulated in a stiff cross‐linked hyaluronic acid hydrogel, showed reduced cell spreading and nuclear localization of YAP/TAZ.^23^ These results indicate that mechanotransduction signaling in a 3D environment is not merely regulated by bulk stiffness, but is sensitive to other parameters such as dimensionality and degradability of the gel.

#### 2.1.2 Local Microenvironment

**Figure 3:**
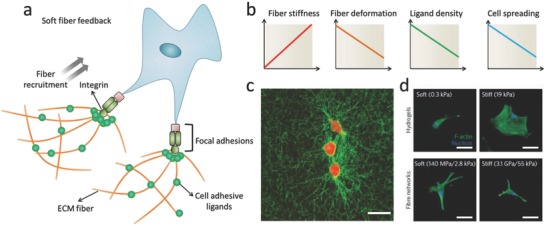
Cell response to local fiber stiffness. a) Schematic image shows how cells response to soft fibers. b) Soft local stiffness increases ability of cells to deform and recruit fibers, thus enhancing concentration of local ligand and activating FAs and related cellular signals to induce cell spreading. c) Cells deform collagen fibers to form bundled structure. Reproduced with permission.^24^ Copyright 2017, Nature Publishing Group. d) Cells failed to spread on substrate with low bulk stiffness, in contrast, increasing fiber stiffness suppressed cell spreading. Reproduced with permission.^25^ Copyright 2015, Nature Publishing Group.

Since the local microenvironment is quite different from the bulk ECM, researchers have started to realize the importance of the local microenvironment of cells. Unlike bulk stiffness, where increased stiffness always promotes cell spreading, materials with soft local stiffness have greater flexibility in changing their conformations to optimize cell contact, and thereby inducing the formation of FAs and relevant cellular signals to trigger cell spreading (**Figure** **3**a). If the fiber stiffness is higher, the transfer of cellular traction forces to nearby fibers will be limited. Consequently, cells are not able to build up sufficient tension, which may suppress cell spreading and migration (Figure 3b). The fibrous nature of the ECM creates a unique microenvironment that enables long range mechanical cell–cell communication via cell‐induced remodeling of the network (Figure 3c).^24^, ^26^ Recently, Baker et al. fabricated a synthetic fibrous material with tunable fiber mechanics by using electrospinning. They found a critical role for fiber recruitment in the cellular response to fibrous materials, where lower fiber stiffness promoted cellular tension to deform and recruit surrounding fibers, greatly increasing the ligand density around the surface of cells, facilitating the formation of FAs and subsequent signaling events (Figure 3d).^25^

Cells are capable of sensing and responding to local mechanical properties in a 3D microenvironment. Recent efforts have focused on producing collagen materials with tunable properties. By controlling the collagen gelation temperature, collagen hydrogels of different fiber stiffnesses can be prepared.^27^ Collagen fiber bundling and diameter can be increased by decreasing the gelation temperature, which results in increasing local fiber stiffness. It was shown that increased local fiber stiffness can withstand the repetitive contractile pulling at cell adhesion sites, which reinforces the stability of cellular adhesion and maturation of human foreskin fibroblasts.[[qv: 27c]] By adding gold nanorods into collagen hydrogels, the nanoscale stiffness of the collagen hydrogel can be tuned without changing the bulk mechanical properties, and increased local collagen stiffness was shown to upregulate β1‐integrin‐mediated signaling pathways.^28^ These emerging insights into how cells respond to local stiffness rather than bulk stiffness have critical implications for the development of new biomaterials for engineering the cell microenvironment in 3D.

#### 2.1.3 Strain‐Stiffening

**Figure 4:**
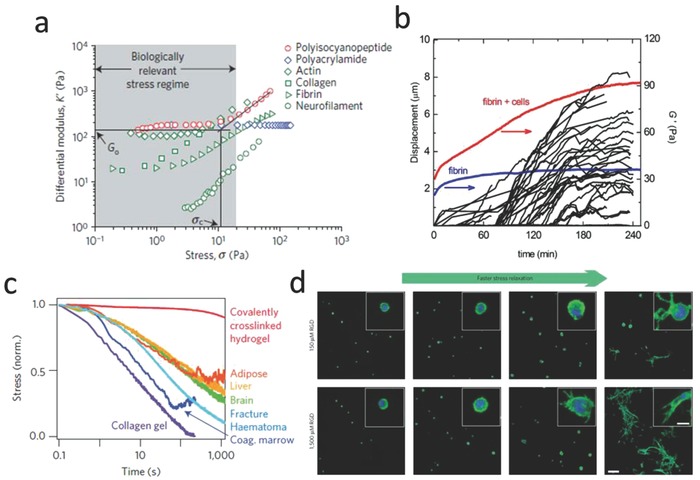
Nonlinear mechanical properties determine cell fate. a) An overview of strain‐stiffening properties for different materials. Reproduced with permission.^29^ Copyright 2016, Nature Publishing Group. b) Cells can generate cell traction forces to actively stiffen fibrin gel. Reproduced with permission.^30^ Copyright 2013, Cell Press. The traction strain was quantified by measuring the displacements of embedded fluorescent beads inside the fibrin hydrogel (black lines). The elastic modulus was measured by real time rheology (red line). The blue curve shows elastic modulus for a pure fibrin gel without cells. c) An overview of stress‐relaxation properties for different materials (including hydrogels and tissues). Reproduced with permission.[[qv: 6b]] Copyright 2016, Nature Publishing Group. d) Hydrogels with faster stress relaxation property can promote cell spreading and proliferation. Reproduced with permission.[[qv: 6b]] Copyright 2016, Nature Publishing Group.

Many filamentous biopolymers (fibrin, F‐actin, microtubules, or vimentin) display nonlinear elasticity, typically strain stiffening (when the applied strain to the matrices is increased beyond the critical strain, the materials become stiffer with increasing strain) (**Figure** **4**a). However, the effects of nonlinear elasticity mechanical properties on cell behavior have barely been studied. Recently, it was shown that hydrogels with nonlinear elasticity facilitate long‐distance communication between cells,^31^ regulate the ways of 3D cell migration,^32^ and control stem cells differentiation.^29^ For example, Janmey and co‐workers demonstrated that fibroblasts and hMSCs displayed an elongated morphology when cultured on soft fibrin gels, indicating that the gels can be deformed by cell traction force, allowing access to the high strain moduli in the regimes of strain stiffening.^31^, ^33^ Shear rheology measurements showed that the cells increased the stiffness of the fibrin gels (Figure 4b).^30^ In a recent study, hydrogels based on the polyisocyanopeptide (PIC) were produced with precisely controlled strain‐stiffening behavior. The critical strain of the PIC hydrogels was increased by increasing the PIC polymer chain length, while the adhesion‐ligand density and the stiffness of PIC bulk hydrogel were kept constant. When cells were cultured in 3D PIC hydrogels, hMSCs preferred to differentiate into osteoblasts when the critical strain was increased, a process apparently mediated by microtubule‐associated protein DCAMKL.^29^ Taken together, these results highlight the strain‐stiffening property as an important element in fabricating 3D microenvironments.

#### 2.1.4 Stress Relaxation

The natural ECM is not an ideal elastic solid. Most hydrogels and soft tissues that are based on biopolymers display viscoelastic (or dissipative) properties.^34^ These hydrogels show stress relaxation (the stress decreases in response to the constant applied strain with increasing time) or creep (the strain increases in response to the constant applied stress with increasing time).^35^ Figure 4c shows stress‐relaxation tests for different materials, including hydrogels and native tissues. Living tissues all exhibit stress relaxation behavior. However, the effects of stress relaxation properties on cell behavior have often been overlooked. In recent years, a number of groups have designed hydrogels with tunable stress relaxation properties by changing the hydrogel composition or concentration,[[qv: 35b]] molecular weight,^36^ cross‐link type or density,^37^ and degradation.^38^ Recent studies demonstrated that hydrogel stress relaxation properties could have significant effects on cell fate decisions. For example, Cooper‐White and co‐workers found that hMSCs morphology, proliferation, and differentiation were influenced by modifications to substrate creep.^39^ Chaudhuri and co‐workers prepared alginate matrices with controllable viscoelastic or elastic features through covalent or ionic cross‐linking, and it was found that when hMSCs encapsulated in the 3D alginate hydrogels with faster relaxation properties, showed enhanced spreading, proliferation, and osteogenic differentiation (Figure 4d). It is thought that integrin signaling, ECM ligand bundling, cell contractility, and nuclear YAP localization all play a role in these processes.[[qv: 6b,40]]

Since most biopolymers show both stress‐relaxation and strain‐stiffening properties, it should be noted that changes in viscoelasticity and nonlinear elasticity are often coupled, which makes it difficult to decouple the two. Chaudhuri and co‐workers found that collagen and fibrin hydrogels exhibited both stiffening and faster stress relaxation upon increasing the strain, an effect attributed to the dissolution of weak cross‐links that are dependent on the force.^41^ Thus, future studies are needed to engineer the 3D cell microenvironment with purely nonlinear elasticity or viscoelasticity behavior, and explore potential applications of these hydrogels in tissue engineering and regenerative medicine.

#### 2.1.5 Surface Receptors

Several recent papers have argued that mechanical feedback of the linkage between ECM substrate and cell surface receptors could influence cell adhesion, spreading, and differentiation.^42^ For example, Trappmann et al. found that cell spreading and differentiation were unaffected by the stiffness of polydimethylsiloxane (PDMS) substrates, but were strongly dependent on the modulus of polyacrylamide (PAAm). The authors proposed that soft PAAm hydrogels were more porous than stiff gels and this will lead to differences in anchoring densities, thereby altering the mechanical feedback of the collagen.[[qv: 42b]] Recently, Navajas and coworkers developed a hydrogel with precisely controlled rigidity and nanometer‐scale distribution of ECM ligands.[[qv: 42e]] They found that when cells were cultured on low‐rigidity substrates, FAs formation could be upregulated by increasing the spacing between ligands, while on high‐rigidity substrates, adhesion collapsed. Moreover, disordered ligand distribution on the substrates significantly increased the stability of adhesion formation, but reduced the rigidity threshold for adhesion collapse.[[qv: 42e]] On the one hand, these results show that the precise nature of the mechanical properties of the link between cells and the substrate must be taken into account when designing substrates for regulating cell fate. On the other hand, cells are very complex systems, and how exactly insoluble physical cues from the cellular environment affect cell behavior still poses a considerable challenge.

#### 2.1.6 Degradability

Many natural materials, such as collagen or fibrin hydrogels, are enzymatically degradable, enabling cells to degrade and remodel their microenvironment. The effect of degradation has a significant effect on cell behavior, especially in 3D microenvironments. Lutolf et al.^43^ highlighted the importance of matrix degradability in studies of cellular invasion into degradable and adhesive synthetic hydrogels. Khetan and Burdick^44^ have shown that cell spreading was limited in hydrogels with a high density of nondegradable cross‐links. They further demonstrated that in 3D covalently cross‐linked hyaluronic acid hydrogels, the differentiation of hMSCs was regulated by the generation of degradation‐mediated cellular traction force, independent of matrix mechanics or cell morphology.^45^ Burdick and co‐workers recently investigated the effect of degradation and stiffness on neural progenitor cell stemness in a 3D hydrogel. The hydrogel was made from elastin like protein and functioned with cell‐adhesive peptide. By changing the protein concentration and cross‐linking density, the stiffness and degradability of hydrogels could be independently tuned. They found that neural progenitor cell stemness did not depend on gel stiffness, but strongly related with degradability. Degradability could increase cell‐mediated matrix remodeling and then enhance neural progenitor cell self‐renewal and potency. This study provided an evidence for the important role of degradability in maintaining neural progenitor cell in 3D microenvironments.[[qv: 4b]] Overall, these results highlight the important role of degradability in regulating cell fate. It should be noted though that controlling the degradation kinetics and the formation of degradation byproducts remains challenging, especially since degradation leads to softening of the ECM, and thus making it harder to present cells with ECM of the right stiffness.

#### 2.1.7 Confinement

**Figure 5:**
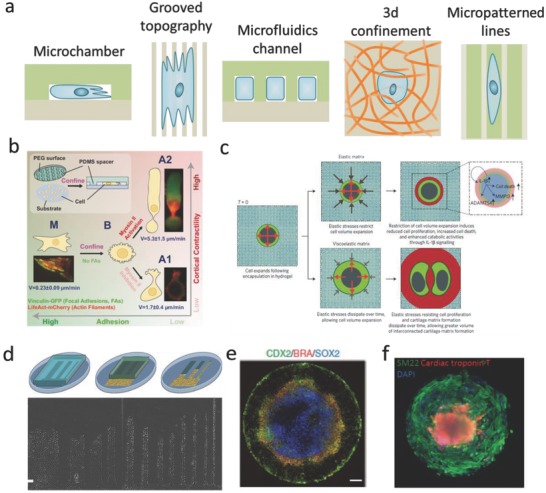
The effect of confinement on cell behavior. a) Schematics of engineered models of confining microenvironments. b) Cells migrate fast in confined environments because of low adhesion. Reproduced with permission.^46^ Copyright 2015, Cell Press. c) Mechanical confinement regulates cartilage matrix formation by chondrocytes. Reproduced with permission.^47^ Copyright 2017, Nature Publishing Group. d) Confinement affects cell migration. Reproduced with permission.[[qv: 22a]] Copyright 2012, National Academy of Sciences (United States). e) Confinement environment is sufficient to induce patterned differentiation of embryonic stem cells. Reproduced with permission.^48^ Copyright 2014, Nature Publishing Group. f) Geometric confinement induced self‐organizing human cardiac microchambers. Reproduced with permission.^49^ Copyright 2015, Nature Publishing Group.

Cells in the body are confined by other cells or by components of the ECM. Therefore, studying the cellular response to confinement is very important for fundamentally understanding the interactions between cells and the ECM. Recently, a lot of in vitro models, including microchambers, grooved substrates, microfluidic channels, microcontact printed substrates, and 3D hydrogels, have been engineered to study the effect of confined environment on cell spreading, migration, and signaling[[qv: 22a,b,46–50]] (**Figure** **5**a).

Cell confinement has been used in a number of different studies, including, for example, studies into the relationship between cell cytoskeleton and cell polarity[[qv: 22b,51]] and cell migration under confinement.[[qv: 50c,51a,52]] On 2D substrates, cells can form distinct FAs and stress fibers to spread and migrate. Conversely, cells in confined environments typically show fewer FAs and suppressed stress fiber formation.[[qv: 51a]] Furthermore, cytoskeletal structures and nuclear elongation are aligned with the confining axis. For example, actin accumulation and stress fibers formation were suppressed under confinement environment, regardless of substrate stiffness.[[qv: 22b,51,52d]] Confinement can also alter the type and morphology of cell adhesions. The homogeneous expression of phosphorylated focal adhesion kinase (pFAK) and p‐paxillin will be inhibited under increased confinement.[[qv: 51a]] Similarly, when cells are limited to 1D fibronectin lines that are generated by microcontact printing, FAs will be distributed along the cell body.^53^ Vinculin will be also homogeneously dispersed over the cell body in cells that are vertically confined^46^ (Figure 5b). By culturing cells in 3D hydrogels, Lee et al. found that when chondrocytes were cultured in hydrogels with slower stress‐relaxation, cell volume expansion was limited by the spatial confinement, resulting in lower cell proliferation rate (Figure 5c).^47^ The influence of confinement on cell migration behavior has also been extensively studied. Cells migration in confinement is typically straight (Figure 5d),[[qv: 22a]] and migration speed is significantly higher in microchannels than on 2D substrates.[[qv: 51a,54]] Fully confined cells display a sliding migration,[[qv: 46,51a,55]] but it remains unclear whether vertical and lateral (confinement affects cell migration equally). Geometric confinement can also influence stem cell differentiation. For example, when human embryonic stem cells colonies were geometrically confined on circular Matrigel micropatterns, they reproducibly differentiated into an outer trophectoderm‐like ring, an inner ectodermal circle, and a ring of mesendoderm that expresses primitive‐streak markers (Figure 5e).^48^ Ma et al. exploited confinement conditions to link spatial cell‐fate specification and the formation of a beating 3D cardiac microchamber, which can be used to mimic certain aspects of early stage heart development (Figure 5f).^49^ Taken together, these studies clearly show that confinement gives rise to marked changes in the cellular cytoskeleton structure, cellular adhesion distributions, cell migration behavior, and stem cell differentiation, indicating that cells are responsive to physical confinement.

#### 2.1.8 Geometrical Cues

**Figure 6:**
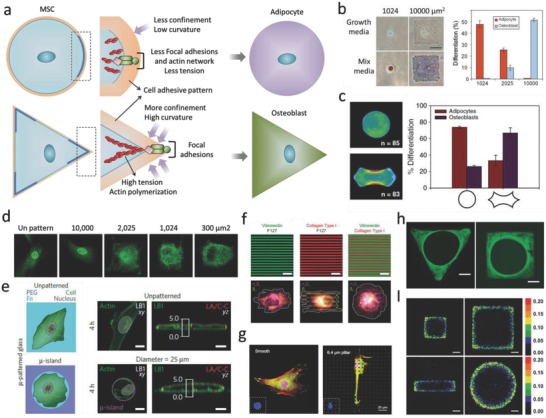
The effect of geometry and topography on cell fate decisions. a) Schematic image shows how cells sense sharp curvature. b) Cell spreading area determines stem cell differentiation. Reproduced with permission.^56^ Copyright 2004, Cell Press. c) Differentiation of hMSCs is determined by cell contractility triggered by different geometries. Reproduced with permission.^57^ Copyright 2010, National Academy of Sciences (United States). d) Cell spreading area directs YAP/TAZ localization. Reproduced with permission.^18^ Copyright 2011, Nature Publishing Group. e) Cell spreading area determines nuclear lamin localization. Reproduced with permission.^19^ Copyright 2015, Nature Publishing Group. f) Substrates with spatially organized multiple adhesive ligands patterns can be used for investigating the effect of various integrin bindings on cell adhesion and migration. Reproduced with permission.^58^ Copyright 2011, Royal Society of Chemistry (United Kingdom). g) With the increase of pillar height, nucleus was deformed, FAs and actin cytoskeletons were densely distributed around the micropillars and became obscure. Reproduced with permission.^59^ Copyright 2016, Elsevier. h) Geometry determines tissue growth rate. Reproduced with permission.^60^ Copyright 2008, The Royal Society (United Kingdom). i) Geometric cues affect cell proliferation rate. Reproduced with permission.^61^ Copyright 2005, National Academy of Sciences (United States).

In native tissue, different cell types vary greatly in their size and shape, and these geometrical cues are important factors in cell fate regulation. The influence of these cues can be studied by culturing cells on micropatterned ECM (for example, collagen, fibronectin, lamin, Matrigel) islands of defined geometries, which can be fabricated with various techniques, for example, microcontact printing/stamping, microwells with different geometries and sizes, and cell printing. When culturing cells on these 2D ECM islands, the cells generate tension forces, and spread until they arrive at the island perimeter.^62^ Cells prefer to generate larger tension at curvature, partially because of the confinement,^63^ and this will lead to upregulation of FAs and actin formation (**Figure** **6**a). The molecular mechanism of cell‐geometry‐dependent regulation of differentiation has been elucidated in some cases.^64^ A recent study suggested that cell geometry regulates cell signaling via modulation of plasma membrane order. Changes in plasma membrane order due to geometric cues affect stem cell fate through a newly identified signaling mechanism involving the serine/threonine kinase Akt/protein kinase B.^65^ Studies on cell geometry have shown that cell fate can be guided between apoptosis, growth, and differentiation by altering the extent to which the cell can physically expand and flatten (Figure 6b).^56^, ^66^ Recent studies demonstrated that the differentiation of MSCs could be switched between osteoblast and adipocytes in a shape‐dependent manner (Figure 6c),^57^ which is partially dependent on the localization of YAP/TAZ (Figure 6d).^18^, ^67^ Cell geometry also plays a very important role in nuclear events. It has been shown that confining cells on patterned surfaces could significantly alter the structural organization of the nuclear lamina compared with cells on flat surfaces (Figure 6e).^19^ Substrate topography (e.g., grooves, steps, pits, etc.) also strongly controls MSC shape and lineage selection. For example, Desai et al. fabricated a substrate with spatially organized multiple adhesive ligands patterns, and found that cells can sense surface geometry by segregating single integrins on the surface of cells to regulate ECM‐specific binding.^58^ (Figure 6f) Cell geometry can also regulate nuclear geometry, which may generate a new way to control stem cell lineage commitment on the subcellular level^59^, ^68^ (Figure 6g). Apart from single cells, tissue growth is also strongly affected by the geometrical features of the matrix. Human epidermal stem cells seeded on 100 µm diameter circular collagen‐coated disks, self‐assembled into a stratified microepidermis. Like the small islands that accommodate single cells, larger islands with a nonadhesive center still supported microepidermis assembly.^69^

Cells in microtissues detect and respond to radii of curvature and when grown in polygonal channels, new tissue started in the corners (Figure 6h).^60^ The tissue in sharp corners (for example, triangular channel) was thicker than those in square and hexagonal channels, following the decrease of local curvature and indicating that increasing local curvature can increase the rate of proliferation (Figure 6I).^61^ Although the idea of 3D micropatterned systems is not novel, technical limitations of these endeavors have limited the feasibility of studying single cell behavior in 3D microenvironments. Recently, we demonstrate the first method to constrain stem cell size and geometry in a systematic and quantitative manner, by encapsulating cells in 3D hyaluronic acid hydrogel microniches.^70^ This method differs from previous studies on 2D micropatterned substrates and microwells, as it can provide cells with a completely nonpolarized microenvironment of precisely defined volume, and it also allows for rapid acquisition of confocal microscopy images on large numbers of individual cells in identical microenvironments. By using this method, we found that cytoskeletal organization in cells in 3D microniches has a preferred size and geometry. Furthermore, we found that key proteins and mRNA concentrations were diluted in larger cells.

**Figure 7:**
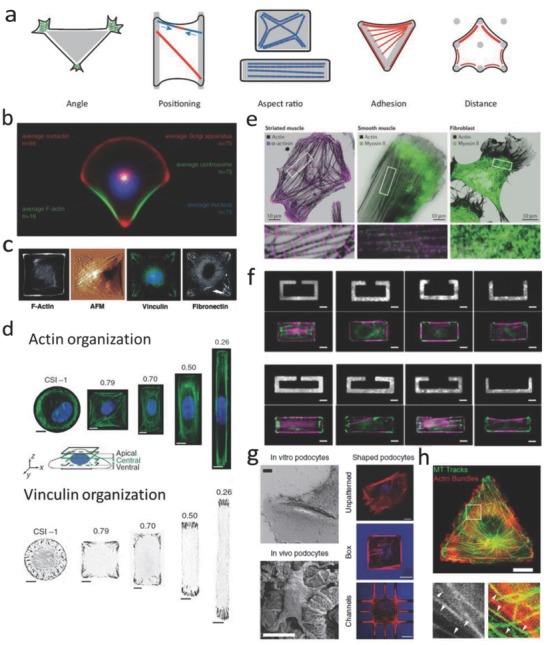
The effect of geometrical cues on actin organization. a) Schematic image showing how geometry directs cytoskeleton organization. Reproduced with permission.^71^ Copyright 2012, Cell Press. b) Organization of polarity is governed by cell adhesive microenvironment. Reproduced with permission.^72^ Copyright 2006, National Academy of Sciences (United States). c) Organization of the stress fibers, FAs, and ECM within cells on a patterned square ECM island. Reproduced with permission.^73^ Copyright 2002, FASEB. d) Cell aspect ratio changes affect organization of actin stress fibers and FAs. Reproduced with permission.^74^ Copyright 2012, Nature Publishing Group. e) Actin, myosin II, and α‐actinin staining for different cell types. Reproduced with permission.^75^ Copyright 2015, Nature Publishing Group. f) Dissipation of elastic energy in severed stress fibers depends on fiber length. Reproduced with permission.^76^ Copyright 2017, National Academy of Sciences (United States). g) (Left) Scanning electron microscopy (SEM) images show in vivo podocytes with branched structure; (Right) F‐actin staining for cells cultured on glass, box, and microchannels. Reproduced with permission.^77^ Copyright 2017, Nature Publishing Group. h) Microtubule growth trajectories are correlated with F‐actin bundles controlled by cell geometry. Reproduced with permission.^78^ Copyright 2012, The Company of Biologists (United Kingdom).

Separate studies show that geometrical cues also affect the orientation of cell motility, initiated by the formation of actin filaments, lamellipodia and filopodia (**Figure** **7**a).^71^ Polarity axes as defined by the internal and cortical cell asymmetry were controlled by the adhesive geometry (Figure 7b).^72^ When cells were cultured on ECM islands with square or rectangle geometry, FAs and actin stress fibers would be inclined to situate along the cell's diagonal axes (Figure 7c,d).^73^, ^74^ The alignment of stress fibers and FAs is partially a result of actomyosin contractility (Figure 7e).^75^ Moreover, it was found that all fibers were connected to each other instead of being isolated and cell relaxation was induced by means of local ablation of one fiber (Figure 7f).^76^ The cell shape within tissue can reflect the past physical and chemical signals that the cells have run into, and the cellular phenotype can also be controlled by the cell shape information. Ron et al. used microfabricated 3D biomimetic chips to demonstrate that 3D cell shape can control cell phenotype via cell tension (Figure 7g).^77^ In addition, it appears that the interplay between actin and microtubuli arrangement plays an important role in cell polarization. In cells spreading on either soft, ECM‐coated gels, or stiff cadherin‐modified substrates, the rearward actomyosin (partially) prevents microtubuli penetration at the leading edge on both soft and stiff substrates.^79^ By contrast, when cells were allowed to spread unconstrained on stiff ECM‐coated substrate, microtubule (MT) aligned in parallel with actin stress fibers, and reached all the way to the leading edge of the cell (Figure 7h).^78^

A range of techniques have been used to control geometric cues on substrates and study their influence on stem cells cultured on such substrates. However, challenges remain. First, it is necessary to assess the influence of geometrical control, after long‐term culture when the cells produce their own ECM and loose direct links with microscale or nanoscale geometrical cues. Second, it remains unclear whether findings on 2D substrates can be applied to 3D. Finally, the underlying molecular mechanisms by which cells sense and respond to the geometric cues, and how the mechanical properties of cells result in the cytoskeleton tension and contractility of cells, are not fully understood.

## 3 Taking Dimensionality into Consideration: From 2D to 3D

As mentioned above, different properties of ECM can be designed to regulate cell fate determination. However, most of these studies involved 2D platforms, which present, by necessity, grossly oversimplified environments compared to the in vivo 3D scenario. In 3D, cells form adhesive connections on all sides, providing an unpolarized environment for cells to grow. The polarized environment and extremely asymmetric distribution of adhesions on 2D substrates may lead to unnatural apical−basal cell polarity and corresponding alterations in cell functions. Besides, cell spreading and adhesion on 2D substrate are unlimited, which allows free spreading and migration of cells without any physical limits. Those fully embedded cells are sterically hindered when they spread and migrate, as they are confined by the surrounding matrix. Cells must penetrate the matrix pores, or degrade the matrix around them, before spreading and migration becomes possible. On 2D substrates, the speed of migration is determined by the actin polymerization, integrin‐mediated adhesion, and myosin‐mediated cellular contraction. However, in a 3D matrix, the contribution effectors to cell migration are very complex, involving, for example, the activation of the nuclear piston,^80^ local ECM stiffness,[[qv: 27c]] membrane tethered protease degradation,[[qv: 27a,81]] the ability to squeeze the nucleus through matrix pores,^82^ and microtubule dynamics.^83^ As a result, the speed of cell migration and its response to stiffness are quite different in 2D compared to 3D. Furthermore, on 2D substrates, cell culture medium, soluble factor, and cell‐secreted factors can undergo free diffusion, whereas in 3D matrices, diffusion of oxygen, proteins, and small molecules can be limited, resulting in gradients.

It is likely that cells cultured in 3D display behavior more relevant to in vivo conditions. Khetan et al. demonstrated that when hMSCs were cultured in covalently cross‐linked HA hydrogels, hMSCs differentiation was controlled by the generation of cellular traction forces mediated by hydrogel degradation, regardless of cell morphology and hydrogel stiffness. These outcomes emphasize the critical role of degradability in 3D as a parameter separate from the influence of cell morphology or substrate.^45^ Recent efforts^84^ on 3D tumor spheroids, aimed at recapitulating the natural tumor microenvironment, showed that 3D tumor spheroids better mimic tumor cell development than traditional 2D monolayer models. Zernicka‐Goetz and co‐workers have shown that by culturing embryonic and extraembryonic stem cells inside a 3D Matrigel, the cells self‐organized into a synthetic embryo, whose development and structure were very similar to those of the natural embryo.^85^

## 4 Technologies to Engineer 3D Stem Cell Niches

**Figure 8:**
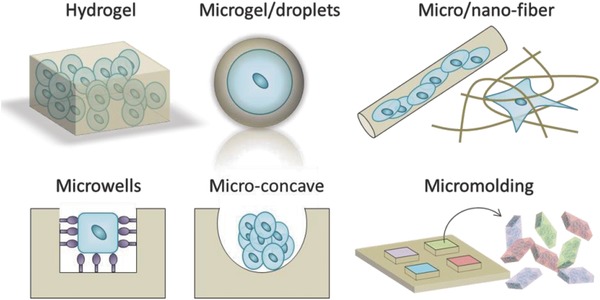
Schematic overview of the major methods used to achieve 3D cell culture.

As discussed above, cells can sense and respond to myriad signals from their 3D microenvironment. Over the past decades, a wide range of sophisticated in vitro cell culture platforms have been developed that control the presentation of biochemical and mechanical cues in 3D. One of the key points to consider in the fabrication of a 3D environment for cells is to allow oxygen and nutrients reach to the compartmentalized cells, while excreted waste products are released. A broad range of fabrication approaches have been employed to control cell–matrix and cell–cell interactions in 3D (**Figure** **8**). In this section, we discuss recent work on bioengineering approaches for controlling interaction between cells and the microenvironment in 3D.

### 4.1 Hydrogel‐Based Technology

Hydrogels, which are water‐swollen cross‐linked polymeric systems, can be prepared from a variety of natural biomaterials and synthetic polymers (**Table** **1**), presenting a wide range of mechanical and chemical features. Many methods can be used to regulate the physical and chemical properties of hydrogels.^2^, ^86^

**Table 1:** Representative materials that can be used for 3D cell culture studies

Materials	Gelation method	Featured properties	Ref.
Natural‐derived materials			
Collagen	Raising the temperature and the pH can initiate collagen fibril self‐assembly	Fibrous structure Exhibits structural and mechanical properties (strain‐stiffening) reminiscent of native tissues Displays native cell adhesion ligands	[[qv: 24,26b,41,51b]]
Fibrin	Thrombin can initiate self‐assembly of insoluble polypeptide chains of fibrinogen into a fibrillar network	Fibrous structure Enzymatically degradable Strain‐stiffening property	30, 93
Gelatin	Gelatin gel can be formed by lowing the temperature or photo‐cross‐linking (for methacrylated gelatin, GelMA)	Stiffness can be controlled Enzymatically degradable	94
Alginate	Alginate hydrogels can be formed by cooperative binding with divalent cations such as Ca^2+^ or Ba^2+^	Should be functioned with adhesive proteins for cell adhesion and spreading Stress‐relaxation property	[[qv: 6b,47,95]]
Hyaluronic acid	Modified HA can form gels by photo‐cross‐linking or enzymatically cross‐linking	It contains a high degree of chemical modification that enables considerable tunability	45, 96
Chitosan	Gels can be formed by adjusting the pH	Excellent biocompatibility and immunostimulatory activities	97
Dextran	Dextran gels can be formed by chemically cross‐linking	Commercially available Cross‐linked dextran can act as a microcarrier	98
Agarose	Cooling initiates the aggregation of double helices by the entanglement of anhydro bridges	Tunable elastic moduli Viscoelastic properties	99
Matrigel	Gels can be formed irreversibly and rapidly between 24 and 37 °C	Gelling speed depends on the concentration and gelation temperature A heterogeneous composition	100
Synthetic materials			
Polyethylene glycol (PEG)	PEG gels can be formed under both physiological pH and temperature	Can be engineered to present different adhesive ligands and to degrade via passive, proteolytic, or user‐directed modes	87
Poly(vinyl alcohol) (PVA)	Modified PVA can form gels under photo‐cross‐linking	Satisfactory biocompatibility and sufficient mechanical properties	101

Naturally derived hydrogels for cell culture are mainly made of proteins and ECM elements, for example, collagen, fibrin, hyaluronic acid, or Matrigel, as well as materials derived from other biological sources such as chitosan, alginate, gelatin, agarose, or dextran.[[qv: 86b,87]] Most of these hydrogels are inherently biocompatible and bioactive, since they are naturally derived.[[qv: 86a]] Some of them (for example, collagen, fibrin, and Matrigel) have binding sites for cells to interact with, and such interactions have some benefits for the viability, proliferation, cell migration, differentiation, and remodeling of the gel matrix.^88^ However, hydrogels made from those natural materials have some disadvantages in isolating certain cell responses and determining exactly which signals are promoting cellular function. For example, Matrigel is comprised of entactin, laminin, and collagen, but also contains a variable and uncharacterized fraction of growth factors.^89^ Furthermore, it is difficult to independently tune the physical and chemical properties for these natural hydrogels.[[qv: 89b]] For example, there is no way to regulate the stiffness of collagen or fibrin gels without changing the adhesive ligand density, pore size, and porosity of the hydrogel. Finally, the shape and size of individual cells cannot be controlled inside hydrogels, and we cannot use hydrogels to make direct comparisons with the outcomes on 2D substrates.

Alternatively, hydrogels composed of synthetic polymers, for example, PEG, can be used for long term cell culture, and allow for ECM deposition as they degrade, suggesting that synthetic gels can be used as 3D cell culture platforms, even when there is no integrin‐binding ligands. Hydrogels made from those synthetic materials are highly reproducible, the mechanical properties can be easily adjusted, and can be conveniently processed. However, they lack the endogenous factors that facilitate cell behavior. These synthetic scaffolds offer a minimalist approach with which the mammalian cells can be cultured in vitro for the purpose of clinical applications and the basic researches of cell physiology.

The ECM is a very dynamic system. To properly mimic the native ECM, some of its complexity (for example, dynamics) must be taken into consideration when designing these hydrogels. Recently, instead of mimicking the static aspects of the cellular microenvironment, researchers started to adopt more dynamic hydrogels. External stimuli can be used to change the chemical and physical properties of hydrogels to better mimic the dynamic native cellular microenvironment. For instance, mechanically dynamic hydrogels that can be stiffened,^90^ softened,^91^ or reversibly stiffened and softened,^92^ have been developed to investigate the effect of stiffness changes on cellular responses. These mechanically dynamic substrates enable us to study the effect of mechanical dosing on cell fate decisions, which is of particular interest for the mechanobiology community.

### 4.2 Microwell‐Based Technology

**Figure 9:**
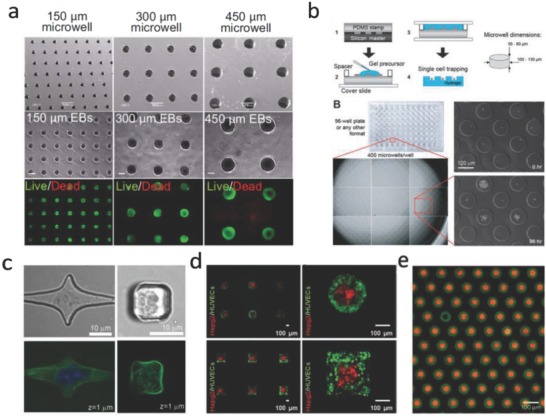
Microwells in cell biology studies. a) ESCs cultured in PEG microwells with different diameters for 7 d. Reproduced with permission.[[qv: 50b]] Copyright 2009, National Academy of Sciences (United States). b) High‐throughput platform based PEG microwells for investigating single cell fate. Reproduced with permission.^102^ Copyright 2009, Royal Society of Chemistry (United Kingdom). c) Confocal images show cells cultured in PDMS microwells with different shapes. Reproduced with permission.^103^ Copyright 2007, Royal Society of Chemistry (United Kingdom). d) Controlling spatial organization of multiple cell types in microwells with certain 3D geometries. Reproduced with permission.^104^ Copyright 2012, Wiley. e) Microwells can be used for creating microparticle arrays with complex building blocks, green particles are assembled before red particles. Reproduced with permission.^105^ Copyright 2017, Nature Publishing Group.

Microwells are a widely used and simple platform to structurally engineer the 3D cell microenvironment. Microwell arrays can be produced by means of direct etching into silicon, or by photolithography, or through molding of hydrogel materials using soft‐lithography. Many different cell types (such as human hepatoblastoma cells, fibroblasts, adipose‐derived stem cells, embryonic stem cells)[[qv: 50b,106]] can be cultured in microwells to form cell spheroids in a high‐throughput manner. For example, embryonic stem cell aggregates can be formed inside microwells of different sizes (**Figure** **9**a).[[qv: 50b]] People found that cardiogenesis was enhanced in larger embryoid bodies (for example, 450 µm in diameter), while the differentiation of endothelial cells was increased in smaller embryoid bodies (for example, 150 µm in diameter). These cell spheroids can be taken as components for bottom‐up tissue engineering applications or serve as efficient 3D in vitro models for research on drug toxicity or cancer invasion. Lutolf et al.^102^ modified and functionalized inside surfaces of microwells with different biomolecules to examine in vitro self‐renewal of hematopoietic stem cells as well as the regulation of this process by recombinant protein signals (Figure 9b). Furthermore, cell density, porosity, and mechanics of the hydrogel as well as the concentration of coated ECM components can be combinatorially regulated in these microwells, which enables a study on the effect of cell–cell interactions as well as hydrogel stiffness on the fate of MSCs.^107^

Changing the sizes and geometric features of microwells can provide tunable confined spaces for controlling cell differentiation. Moreover, by culturing cells in microwells, the influence of cell shape, substrate stiffness, and dimensionality can be decoupled (Figure 9c). For example, Tsurkan et al.^108^ fabricated microwells and microchannels with defined architectures using microlens array photopatterning technology, and they identified that neural precursor cell differentiation is dependent on the degree of spatial confinement. However, most reported microwell cultural systems are immobile, limiting their possibilities to actively operate encapsulated individual cells. Recently, microwells with varied dynamically adjustable geometries have been designed by using biocompatible polymers that are responsive to temperature, such as polycaprolactone (PCL).^109^ The dynamic alterations in microwell geometries resulted in dramatic changes in the cytoskeletal architecture and differentiation patterns of stem cells. Tekin et al. prepared dynamic microwells with tunable shape transformation properties under different temperatures by using poly(*N*‐isopropylacrylamide) (PNIPAAm), a thermoresponsive polymer. This feature was exploited to pattern multiple cell types at different temperatures in dynamic circular and square microwells^104^ (Figure 9d).

Cellular microwell arrays can provide high throughput platforms for deconstructing the multicomponent cues that regulate cell function, and can be used to create large‐scale microparticle arrays with complex motifs^105^ (Figure 9e). However, the limitation of using microwells for cell culture is that they are pseudo‐3D models that cannot really mimic the in vivo 3D environment; therefore, more advanced and integrative technologies should be developed to engineer the biophysical microenvironment of cells.

### 4.3 Microgel‐Based Technology

Inspired by observing different organs or tissues that consist of repetitive building blocks (think of hepatic lobules or nephron architecture), microgels have been fabricated for 3D cell encapsulation. To date, microgels have been fabricated with different shapes and sizes by using different methods. For example, a patterned photomask could be used to fabricate microgels with an array of shapes. By expanding this method, Fan et al.^110^ presented a two‐step method based on photolithography technology to encapsulate single neuron cells in gelatin microgels, and found that axonal circles formed in these hydrogel rings mimicking self‐synapse diseases. Another common approach to create microgels involves the use of a micropatterned mold. For example, by using a patterned PDMS stamp, HA microgels containing the cells could be molded under UV cross‐linking.^111^ By using the same method, more complex 3D cell microenvironments over multiple size scales can be fabricated.^112^ Recently, Ma et al. engineered a hyaluronic acid microgel that contains fibrinogen by using droplet‐based microfluidics.^113^ The microgels serve as a 3D microenvironment for culturing of single hMSCs, and could be cultured up to 4 weeks with different stiffness (0.9–9.2 kPa).^114^ One recent study from Weitz and co‐workers^115^ shows that by using microfluidic technology, single cells could be encapsulated in 3D alginate microgels, and cells remained viable in microgels over three days. It was found that the osteogenic differentiation of encapsulated cells was determined by the cell density or gel stiffness, and the work also demonstrated that by injecting the singly encapsulated marrow stromal cells intravenously into the mice, the clearance kinetics were postponed and the donor‐derived soluble factors in vivo were maintained. Therefore, encapsulation of individual cells in microgels might be useful in the field of regenerative medicine applications and tissue design.

## 5 Conclusions and Outlook

The use of engineered 3D cellular microenvironments enables us to look into the way in which cells interact with and react to the external environment. However, much work remains to be done. Cells are rarely in equilibrium, and understanding how cells accumulate information about their environment over time, how external stimuli are translated molecularly into cell face decisions, and how these decisions manifest themselves in changes in cell phenotype remain core questions for cell biology. Controlling the environment as much as possible can help answer these questions. To provide a very small glimpse, we recently found that cell spreading dynamics could provide a strong indication of future cellular behavior.^116^ Future work should focus on developing new ways to track and observe single cell dynamics over extended periods of time, while building up a molecular picture of the changes occurring in the cell.

It should be noted that changes do not only occur inside cells, cells also modify their surroundings. A very promising development there is the engineering of biomimetic materials with time‐regulated properties that react to external stimuli.^90^, ^92^, ^117^ However, most of these dynamic materials only result in mechanical or topographical changes, which is oversimplified when compared to the in vivo cell microenvironment dynamics. Therefore, future work should focus on developing new materials that allow the real‐time control of cell microenvironments and fully capture cell dynamics. Ultimately, we need all of this information to understand how we can engineer synthetic microenvironments for developing and maintaining living tissues inside synthetic compartments.

## Conflict of Interest

The authors declare no conflict of interest.
